# The Value of Mobile Health in Improving Breastfeeding Outcomes Among Perinatal or Postpartum Women: Systematic Review and Meta-analysis of Randomized Controlled Trials

**DOI:** 10.2196/26098

**Published:** 2021-07-16

**Authors:** Jiafen Qian, Tingting Wu, Meina Lv, Zongwei Fang, Mingrong Chen, Zhiwei Zeng, Shaojun Jiang, Wenjun Chen, Jinhua Zhang

**Affiliations:** 1 Department of Pharmacy Fujian Medical University Union Hospital Fuzhou China; 2 College of Pharmacy Fujian Medical University Fuzhou China

**Keywords:** mHealth, breastfeeding, randomized controlled trial, meta-analysis

## Abstract

**Background:**

Breastfeeding is essential for maintaining the health of mothers and babies. Breastfeeding can reduce the infection rate and mortality in newborns, and can reduce the chances of overweight and obesity in children and adolescents. For mothers, a longer duration of breastfeeding can reduce the risk of breast cancer, ovarian cancer, and type 2 diabetes. Although breastfeeding has many benefits, the global breastfeeding rate is low. With the progress of time, the popularity of mobile devices has increased rapidly, and interventions based on mobile health (mHealth) may have the potential to facilitate the improvement of the breastfeeding status.

**Objective:**

The main objective of this study was to analyze the existing evidence to determine whether mHealth-based interventions can improve the status of breastfeeding.

**Methods:**

We systematically searched multiple electronic databases (PubMed, Web of Science, The Cochrane Library, Embase, CNKI, WanFang, and Vip ) to identify eligible studies published from 1966 to October 29, 2020. Included studies were randomized controlled trials (RCTs) studying the influence of mHealth on breastfeeding. The Cochrane Collaboration Risk of Bias tool was used to examine the risk of publication bias. RevMan 5.3 was used to analyze the data.

**Results:**

A total of 15 RCTs with a total sample size of 4366 participates met the inclusion criteria. Compared with usual care, interventions based on mHealth significantly increased the postpartum exclusive breastfeeding rate (odds ratio [OR] 3.18, 95% CI 2.20-4.59; *P*<.001), enhanced breastfeeding self-efficacy (mean difference [MD] 8.15, 95% CI 3.79-12.51; *P*=.002; I^2^=88%), reduced health problems in infants (OR 0.62, 95% CI 0.43-0.90; *P*=.01; I^2^=0%), and improved participants’ attitudes toward breastfeeding compared with usual care (MD 3.94, 95% CI 1.95-5.92; *P*<.001; I^2^=0%). There was no significant difference in the initiation of breastfeeding within an hour of birth between the intervention group and the usual care group (OR 1.26, 95% CI 0.55-2.90; *P*=.59). In addition, subgroup analysis was carried out according to different subjects and publication times. The results showed that the breastfeeding rate was not limited by the types of subjects. The breastfeeding rate based on mHealth at 1 month and 2 months after delivery did not change over the time of publication (2009 to 2020), and the breastfeeding rate based on mHealth at 3 months and 6 months after delivery gradually increased with time (2009 to 2020).

**Conclusions:**

Interventions based on mHealth can significantly improve the rate of postpartum exclusive breastfeeding, breastfeeding efficacy, and participants’ attitudes toward breastfeeding, and reduce health problems in infants. Therefore, encouraging women to join the mHealth team is feasible, and breastfeeding-related information can be provided through simple measures, such as text messages, phone calls, and the internet, to improve the health of postpartum women and their babies.

## Introduction

Proper feeding is a prerequisite for the healthy growth of babies. The World Health Organization (WHO) recommends starting exclusive breastfeeding within an hour of birth and continuing it for at least 6 months after delivery. However, maintaining breastfeeding to 2 years or longer can be beneficial to the health of both infants and mothers. For babies, early initiation and exclusive breastfeeding within 6 months can reduce the infection rate and mortality in newborns, and continuous breastfeeding for 2 years or longer can reduce the chances of overweight and obesity in children and adolescents. For mothers, a longer duration of breastfeeding can reduce the risk of breast cancer, ovarian cancer, and type 2 diabetes [1].

Although breastfeeding has many benefits, the rate of exclusive breastfeeding within 6 months in low-income countries and middle-income countries is only 37%, and in high-income countries, the duration of exclusive breastfeeding is shorter than that in low-income and middle-income countries [2]. Victora et al reported that a total of 63% of infants younger than 6 months were not breastfed, and the weighted prevalence for 6 months of exclusive breastfeeding was 20.8% [2]. Moreover, the exclusive rate was found to be only 17% in Chinese urban areas [3].

Many factors have been identified as having an impact on breastfeeding outcomes, and a key to solving the problem of the low breastfeeding rate is to improve awareness among pregnant women and mothers, as well as perform regular follow-ups [4]. Face-to-face interventions require high levels of cooperation in postpartum women, and it is easy for women to be lost to follow-up. One proposed solution is mobile health (mHealth), which could provide medical assistance with the help of electronic mobile devices. Compared to face-to-face medical assistance, mHealth is cheaper and can have improved compliance [5]. Thus, mHealth is being applied in an increasing number of fields [5-7]. A new mother’s mood may change from extreme joy to tension and anxiety, which may stimulate her to use electronic mobile devices to search for breastfeeding knowledge. These therefore provide the best entry point for mHealth [8]. Information can be provided by professional medical staff or trained volunteers with breastfeeding experience [8]. Since volunteers are more likely to resonate with primiparous mothers, they may be more suitable to help primiparous women with low income or with basic or no education.

Previous research into the effectiveness of mHealth-based interventions for promoting breastfeeding have been inconclusive. Therefore, the purpose of this study was to integrate the best evidence to clarify whether these interventions can improve the current breastfeeding status.

## Methods

### Search Strategy

A systematic search of databases (PubMed, Embase, The Cochrane Library, and Web of Science) was conducted to identify eligible studies published from 1966 to October 29, 2020. The retrieval strategy of the PubMed database was as follows: ((“breastfeeding” OR “exclusive breastfeeding”) AND (“Mobile Applications” OR “Telemedicine” OR “Text Messaging” OR “Cell Phone” OR “Smartphone” OR “mHealth” OR “eHealth” OR “Mobile” OR “Portable Software Application” OR “Tele*” OR “e-Health” OR “m-Health” OR “?phone*” OR “Text*” OR “Short Message” OR “SMS” OR “App” OR “Apps” OR “App-based” OR “Electronic” OR “Message*” OR “Web” OR “Web-based” OR “Internet*” OR “Digital*”) AND (“randomized controlled trial” OR “controlled clinical trial” OR “randomized” OR “placebo” OR “clinical trials as topic” OR “randomly” OR “trial”) NOT (“animals”) NOT (“humans” AND “animals)). The detailed search strategy for each database is presented in Multimedia Appendix 1. To ensure that the search was comprehensive, we also searched the reference lists of the studies yielded by the original search. This study was performed in accordance with the recommendations of the Preferred Reporting Items for Systematic Reviews and Meta-Analyses (PRISMA) statement [9].

### Study Inclusion Criteria

We included all studies that met the following requirements: (1) research subjects were pregnant or postpartum women; (2) the intervention group included studies that involved mHealth interventions, such as phone calls, text messages, and interactive computer systems, and the control group received usual care; (3) the study was a randomized controlled trial (RCT); (4) the definition of breastfeeding conformed to the WHO definition; and (5) the study mentioned the calculation of sample size and reported enough data to calculate the effect size.

### Study Exclusion Criteria

Studies were excluded from the meta-analysis if (1) both the intervention and control groups accepted mHealth treatment; (2) the data could not be obtained, or the extracted data could be combined with other data; and (3) the study was not published in English.

### Literature Screening and Data Extraction

Literature screening first involved reading the title and abstract to determine if the study met the inclusion criteria and then reading the full text before finally determining whether it should be included. The main data extracted were (1) the name of the first author and the date of publication; (2) research characteristics, such as the mean sample age, interventions, and sample size; and (3) outcomes, including exclusive breastfeeding rate, breastfeeding self-efficacy, health problems of infants, rate of initiation of breastfeeding within an hour of birth, and maternal attitude to breastfeeding. Data extraction was performed independently by two reviewers. Any discrepancies were resolved by discussion or by a third investigator.

### Study Quality Assessment

The bias of RCTs included in the systematic review was assessed using the Cochrane tool [10]. The following indicators of internal validity specific to the methodology of an RCT were collected: (1) random sequence generation, (2) allocation concealment, (3) blinding of participants and personnel, (4) blinding of outcome assessment, (5) incomplete outcome data, (6) selective reporting, and (7) other bias. An additional researcher was asked to conduct an evaluation to help resolve disputes that arose during the evaluation process.

### Statistical Analysis

The meta-analysis was performed using RevMan 5.3. The odds ratios (ORs), mean differences (MDs), 95% CIs, and *P* values were calculated. Statistical significance was considered at a *P* value <.05. The heterogeneity among the included studies was analyzed by the chi-square test, and the test level was α=.10. If *P* was ≥.1 and I^2^ was ≤50%, a fixed effects model was used for the meta-analysis. If *P* was <.1 and I^2^ was >50%, a random effects model was used for the meta-analysis. We also used subgroup analysis to detect the source of heterogeneity and carried out a sensitivity analysis using the method of one-by-one exclusion.

## Results

### Search Results

**Figure 1:**
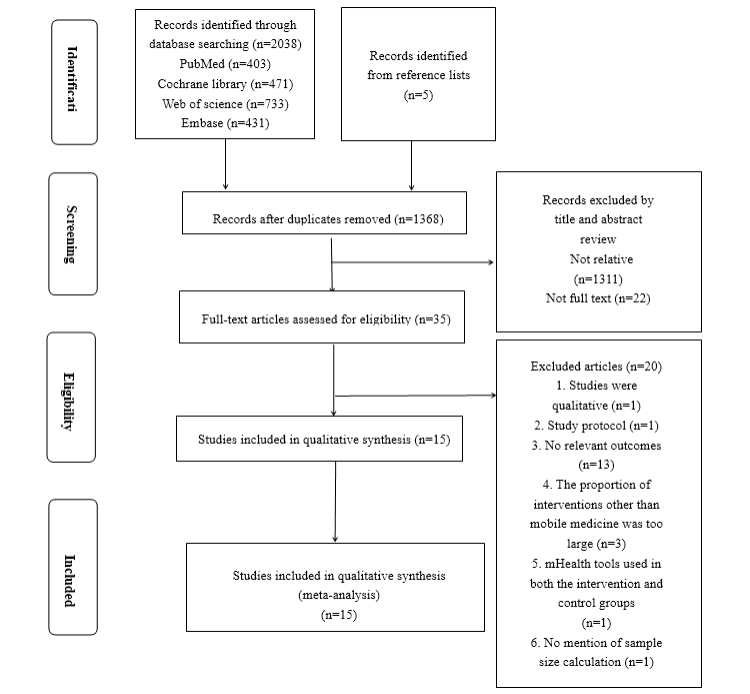
Screening flowchart.

A total of 1368 papers were found, and further screening yielded 35 papers for the full-text search. Of these, 20 papers were excluded owing to irrelevant content, failure to meet the inclusion criteria, and qualitative results. The screening process is shown in Figure 1.

### Study Characteristics

A total of 15 RCTs were included in this study, and the basic characteristics of the included studies are shown in Table 1 [4-8,11-20]. The intervention measures included were divided into (1) telephone support (one article mentioned this intervention), (2) SMS text messaging (one article), (3) internet intervention (seven articles), and (4) telephone, SMS text messaging, and other interventions (six articles). The subjects of 11 studies were pregnant women, and the subjects of four studies were postpartum women. The age of the subjects ranged from 16 to 49 years, and the follow-up duration ranged from 24 hours to 6 months.

**Table 1:** Characteristics of the clinical trials included in this study.

First author and year	Mode of intervention	Location	Type of participant	Intervention subjects, n	Control subjects, n	Outcomes
Sari, 2020 [12]	Web-based program	Turkey	Pregnant women	35	36	1. Infant prevalence
Wen, 2020 [13]	Telephone support + SMS support	Australia	Pregnant women	770	385	1. Exclusive breastfeeding rate
Uscher-Pines, 2019 [11]	Video call	North Central Pennsylvania	Postpartum women	94	93	1. Exclusive breastfeeding rate
Puharic, 2019 [5]	Telephone support + booklet	Split Dalmatia County	Pregnant women	232	123	1. Exclusive breastfeeding rate 2. Infant prevalence 3. ⅡFAS^a^
Cavalcanti, 2019 [4]	Online social network	Northeast Brazil	Postpartum women	123	128	1. Exclusive breastfeeding rate
Patel, 2018 [20]	Telephone support + SMS support + standard management	Rural India	Women in the third trimester	519	518	1. Exclusive breastfeeding rate 2. Infant prevalence
Araban, 2018 [6]	SMS support + courses + standard management	Iran	Pregnant women	56	54	1. Exclusive breastfeeding rate 2. BSES^b^, BSES-SF^c^
Ahmed, 2017 [17]	Breastfeeding monitoring system	Midwestern Hospital	Postpartum women	49	57	1. Exclusive breastfeeding rate 2. Infant prevalence
Efrat, 2016 [8]	Telephone support + standard management	Spain	Pregnant women	111	109	1. Exclusive breastfeeding rate
Flax, 2014 [14]	Telephone support + courses	Nigeria	Pregnant women	196	194	1. Exclusive breastfeeding rate
Bonuck, 2014 [7]	E-prompt	Bronx	First or second trimester of a singleton pregnancy	236	77	1. Exclusive breastfeeding rate
Scott, 2013 [16]	Web-based program	United States	Pregnant women	49	50	1. ⅡFAS
Tahir, 2012 [18]	Telephone support + standard management	Malaysia	Postpartum women	179	178	1. Exclusive breastfeeding rate
Simonetti, 2011 [19]	Telephone support	Italy	Postpartum women	55	59	1. Exclusive breastfeeding rate
Pate, 2009 [15]	Web-based program	United States	Pregnant women	23	23	1. BSES, BSES-SF

^a^ⅡFAS: Infant Feeding Attitude Scale (17-item 5-point scale).

^b^BSES: Breastfeeding Self-Efficacy Scale, a mother’s confidence in her ability to breastfeed.

^c^BSES-SF: Breastfeeding Self-Efficacy Scale-Short Form, a measurement of exclusive breastfeeding self-efficacy (14-item 5-point scale).

### Risk of Bias

**Figure 2:**
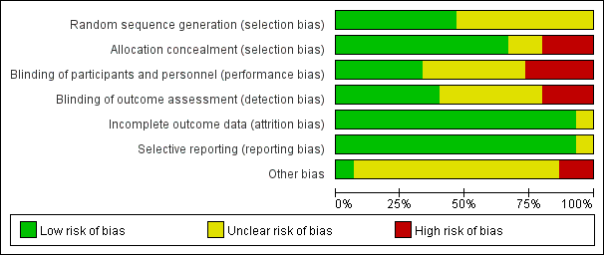
Bias risk assessment chart.

**Figure 3:**
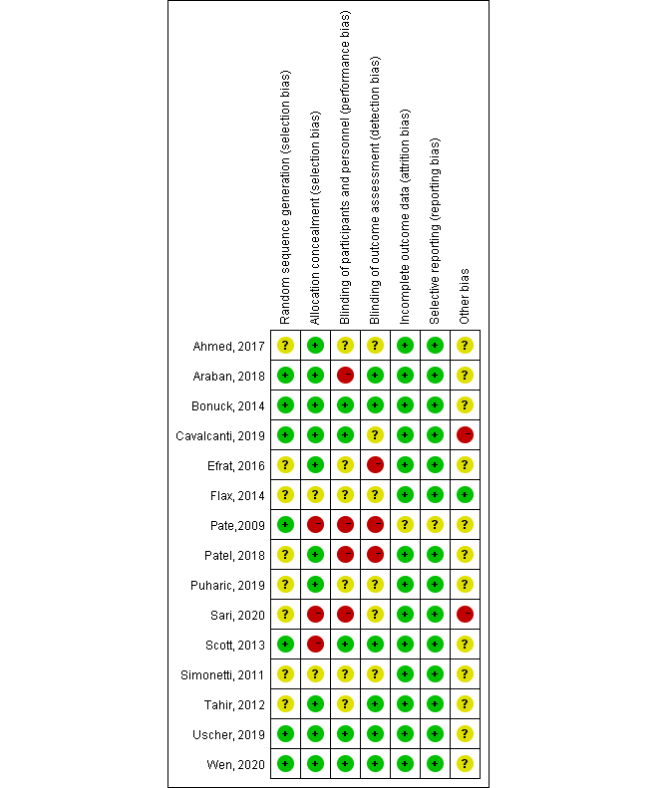
Summary of risk of bias.

The bias of RCTs included in the systematic review was assessed using the Cochrane tool, and the results are shown in Figures 2 and 3. Of the 15 papers included, seven papers did not mention the generation and allocation of random sequences, nine papers did not mention whether the participants and intervention providers were blinded, and five papers did not mention whether the evaluator was blinded. All studies reported the outcome indicators mentioned in the research protocol or method.

### Meta-analysis Results

#### Exclusive Breastfeeding Rate at 1 Month After Delivery

**Figure 4:**
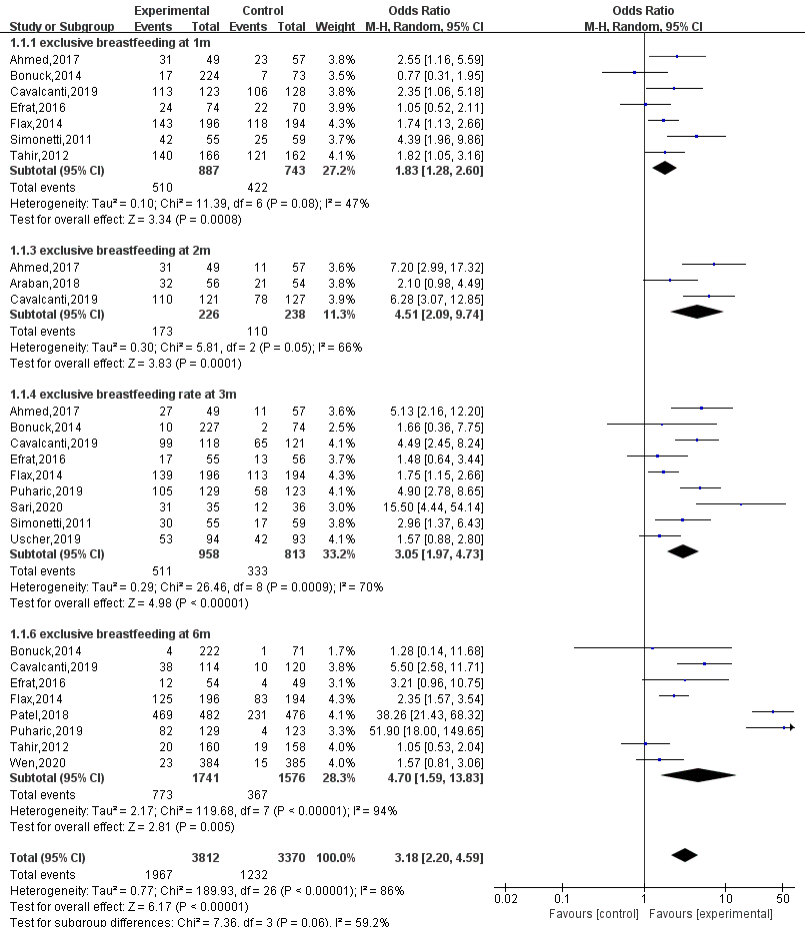
Forest plot of exclusive breastfeeding rates.

A total of seven studies [4,7,8,14,17-19] reported exclusive breastfeeding rates at 1 month after delivery. The results of random effect model analysis showed that mHealth-based interventions significantly improved the rate of exclusive breastfeeding compared with usual care (OR 1.83, 95% CI 1.28-2.06; *P*<.001; I^2^=74%). The sensitivity analysis showed that the results were stable. The forest plot is shown in Figure 4.

#### Exclusive Breastfeeding Rate at 2 Months After Delivery

A total of three studies [4,6,17] reported exclusive breastfeeding rates at 2 months after delivery. The results of random effect model analysis showed that mHealth-based interventions significantly improved the rate compared with usual care (OR 4.51, 95% CI 2.09-9.74; *P*<.001; I^2^=66%). Although there was heterogeneity among the studies, the sensitivity analysis showed that the results were stable. We conducted a subgroup analysis to find heterogeneity from intervention measures, sample size, publication year, types of subjects, etc. The source of heterogeneity was not found, and we inferred that heterogeneity may have been the result of a combination of multiple factors. The forest plot is shown in Figure 4.

#### Exclusive Breastfeeding Rate at 3 Months After Delivery

A total of nine studies [4,5,7,8,11,12,14,17,19] reported exclusive breastfeeding rates at 3 months after delivery. The results of random effect model analysis showed that mHealth-based interventions significantly improved the rate (OR 3.05, 95% CI 1.97-4.73; *P*<.001; I^2^=70%). Although the studies showed heterogeneity, the sensitivity analysis showed that the results were stable. We conducted a subgroup analysis to find heterogeneity from intervention measures, sample size, publication year, types of subjects, and average number of interventions, etc. The source of heterogeneity was not found. The forest plot is shown in Figure 4.

#### Exclusive Breastfeeding Rate at 6 Months After Delivery

A total of eight studies [4,5,7,8,13,14,18,20] reported exclusive breastfeeding rates at 6 months after delivery. The results of random effect model analysis showed that mHealth-based interventions significantly improved the rate compared with usual care (OR 4.70, 95% CI 1.59-13.83; *P*=.005; I^2^=94%). Although there was heterogeneity among the studies, the sensitivity analysis showed that the results were stable. We conducted a subgroup analysis to find heterogeneity from intervention measures, sample size, publication year, types of subjects, average number of interventions, literature quality, etc. The source of heterogeneity was not found, and we inferred that excessive heterogeneity may have been the result of a combination of multiple factors. The forest plot is shown in Figure 4.

### Subgroup Analysis

In order to explore whether a different starting time of the intervention has an effect on the rate of exclusive breastfeeding, a subgroup analysis was carried out according to different types of subjects. The results of the study showed that there was no significant difference between the pregnancy group and the postpartum group for the increase in the rate of exclusive breastfeeding at 1, 2, 3, and 6 months after delivery, indicating that the time to start the intervention had no effect on the increase in the breastfeeding rate. The forest plots are shown in Figures S1-S4 in Multimedia Appendix 2.

We also conducted a subgroup analysis of the publication year. We found that the publication time of the study did not influence the breastfeeding rate at 1 and 2 months after delivery, and the reason may be that people generally think exclusive breastfeeding in the short term after delivery is very important. Therefore, it does not show a significant time effect. However, with extension of the follow-up, the publication time of the study had an impact on the breastfeeding rate. The possible reason is that with the extension of time, people stop exclusive breastfeeding due to lack of corresponding knowledge. However, with the comprehensive popularization of mobile devices in recent years, people’s perceptions have changed in all directions. They are paying more attention to breastfeeding, and there are increasing number of ways to obtain breastfeeding knowledge. Thus, the breastfeeding rate at 3 months after delivery has gradually increased with time. The forest plots are shown in Figures S5-S8 in Multimedia Appendix 2.

### Breastfeeding Self-Efficacy

**Figure 5:**
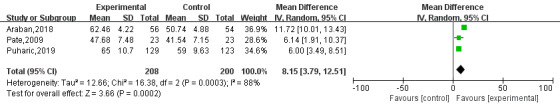
Forest plot of breastfeeding self-efficacy.

**Figure 6:**
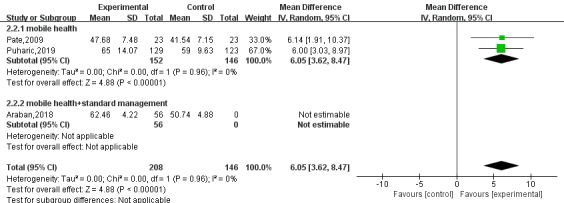
Forest plot of the breastfeeding self-efficacy subgroup.

A total of three [5,6,15] studies reported on breastfeeding efficacy. The results of random effects model analysis showed that mHealth-based interventions significantly improved breastfeeding efficacy compared with usual care (MD 8.15, 95% CI 3.79-12.51; *P*<.001; I^2^=88%). Although there was heterogeneity among the studies, sensitivity analysis showed that the results were stable. Through subgroup analysis of the data, the heterogeneity between the studies was significantly reduced, indicating that the intervention measures may have been the main source of the heterogeneity. The forest plots are shown in Figures 5 and 6.

### Infant Hospitalization

**Figure 7:**
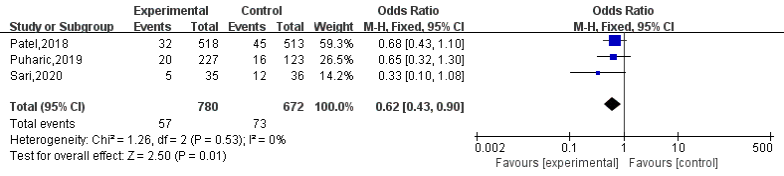
Forest plot of health problems of infants.

A total of three [5,12,20] studies reported the rate of infant health problems. The results of fixed effects model analysis showed that mHealth-based interventions significantly reduced health problems in infants compared with usual care (OR 0.62, 95% CI 0.43-0.90; *P*=.01; I^2^=0%). The sensitivity analysis showed that the results were unstable. The sensitivity analysis was performed by removing the studies one by one. However, after removing the study by Sari et al, there was no significant difference between the intervention and control groups (OR 0.67, 95% CI 0.46-0.99; *P*=.05; I^2^=0%). The forest plot is shown in Figure 7.

### Participants’ Attitudes Toward Breastfeeding

**Figure 8:**
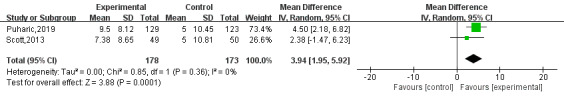
Forest plot of breastfeeding attitudes.

A total of two [5,16] studies reported participants’ attitudes toward breastfeeding. The results of random effects model analysis showed that mHealth-based interventions significantly improved participants’ attitudes toward breastfeeding compared with usual care (MD 3.94, 95% CI 1.95-5.92; *P*<.001; I^2^=0%). The forest plot is shown in Figure 8.

### Initiation of Breastfeeding Within an Hour of Birth

**Figure 9:**
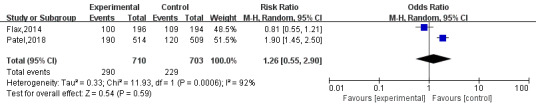
Forest plot of initiation of breastfeeding within an hour of birth.

Two studies [14,20] reported the rate of initiation of breastfeeding within an hour of birth. There was no significant difference in the initiation of breastfeeding within an hour of birth between the intervention group and the usual care group (OR 1.26, 95% CI 0.55-2.90; *P*=.59; I^2^=92%). The forest plot is shown in Figure 9.

## Discussion

### Principal Findings

In this meta-analysis, we included 15 RCTs comprising 4293 patients. The purpose of this meta-analysis was to evaluate whether mHealth-based interventions can improve the current breastfeeding situation compared with usual care. The meta-analysis showed that these interventions could improve the rate of exclusive breastfeeding at 1, 2, 3, and 6 months after delivery, improve breastfeeding efficacy, and reduce health problems in infants. Since breastfeeding efficacy has a great impact on postpartum breastfeeding, using mHealth interventions to enhance breastfeeding efficacy could greatly improve the breastfeeding status. As for breastfeeding attitude and the proportion of rapid initiation of breastfeeding, there was no significant difference between the groups. Thus, interventions based on mHealth are effective for improving the breastfeeding status.

In terms of health problems in infants, sensitivity analysis showed that the results were unstable. This may be related to the inconsistent follow-up duration. One paper assessed the rate over 3 months, one assessed the rate from 3 to 6 months, and one assessed the rate in the first 6 months. It may also be related to the different intervention modes used, which were telephone support and other interventions, telephone and SMS support, and internet-based support.

In terms of the exclusive breastfeeding rate, this study found that mHealth-based interventions increased the rate, and this is consistent with a study by Lee et al [21]. However, our meta-analysis investigated several factors, whereas the previous meta-analysis [21] mainly studied the effect on the health status of mothers and babies. Another meta-analysis [22] assessed the initiation of breastfeeding, breastfeeding efficacy, breastfeeding attitude, and breastfeeding duration, but there were many differences between the two studies. First, our study included 15 RCTs, whereas only six RCTs were included in the previous meta-analysis [22]. Second, our study compared the rate of exclusive breastfeeding from childbirth to 6 months postpartum, allowing the effects of mHealth-based interventions to be directly seen. The previous meta-analysis [22] only compared the duration of exclusive breastfeeding (not intuitive enough). Third, whether mobile medicine can increase the breastfeeding rate within 1 hour of birth has not been found in previous studies. Fourth, the previous meta-analysis [22] showed that mHealth interventions did not improve the efficacy of breastfeeding, whereas our study, which included more RCTs, did find an improvement with the use of mHealth interventions. We therefore conclude that mHealth is very important for promoting breastfeeding. Fifth, we explored whether interventions in different periods have an impact on the results.

The two existing measures to improve the breastfeeding status have their own advantages and limitations. One way to effectively convey health information to mothers who wish to breastfeed is mHealth-based interventions. The verbal and nonverbal communication behaviors of mHealth used by the provider can be used to build trust with the patients to improve satisfaction and adherence to the treatment plan [23]. mHealth can be widely applied to areas with low income and low medical levels in order to reduce medical expenses of postpartum women and improve their attitude toward breastfeeding. Second, it can improve maternal well-being and reduce anxiety by providing maternal and childcare information during pregnancy. With the improvement in health knowledge, maternal mental health may also be improved [22]. Third, fathers can also actively participate in pregnancy and postpartum care through the use of mobile apps. An increase in paternal participation can improve maternal confidence and attitudes toward breastfeeding, which can greatly increase the rate of health care use [24]. Finally, mHealth procedures can be used to collect pregnancy and child health data to facilitate the development of related research [21].

### Limitations

Breastfeeding can not only reduce the risk of breast cancer and ovarian cancer, but also promote the healthy growth of babies [1]. Increasing numbers of mothers are realizing the importance of breastfeeding, but due to a lack of knowledge about breastfeeding, the overall level of breastfeeding in China and foreign countries is slightly low [2,3]. With the development of the economy and society, the popularity of the internet and mobile devices is increasing, which will provide an opportunity to increase the breastfeeding rate. Our research showed that the use of mHealth to convey key breastfeeding information to mothers during pregnancy or after delivery can help increase the breastfeeding rate. Therefore, we can use mHealth to provide pregnant or postpartum women with relevant knowledge and solve the problems that they encounter in the process of breastfeeding, so as to improve breastfeeding confidence and attitude, and achieve an increase in the breastfeeding rate. Women who want to increase the breastfeeding rate can positively seek help from people with knowledge of breastfeeding through telephone, text messages, the internet, and other tools before or after delivery, which can solve the problems that they encounter during breastfeeding and improve breastfeeding self-efficacy and attitude. For example, they can improve self-breastfeeding knowledge by watching breastfeeding videos on the internet or using electronic devices to communicate with breastfeeding professionals about the problems they encounter.

The study has several limitations. First, there was insufficient literature on several outcomes, which may lead to bias. For example, the outcome of rapid initiation of breastfeeding requires more data to obtain more reliable results. Second, the results of the sensitivity analysis of some outcomes were not stable, which may lead to bias, and they need to be further verified by more studies. Third, several articles were not highly representative. For example, the research subjects in the study by Flax et al [14] were women with microfinance, and since this population is not representative, these results should not necessarily be extrapolated to the whole population. Third, the source of heterogeneity needs to be further assessed in future research.

### Conclusion

Our study found that interventions based on mHealth can improve the rate of exclusive breastfeeding, the breastfeeding attitude of mothers, and breastfeeding efficiency, and reduce health problems in infants. In view of the universality of mobile devices, mHealth can be used to promote the health of pregnant mothers and infants. The meta-analysis found limited improvement in rapid initiation of breastfeeding with mHealth interventions. More clinical studies are needed to confirm this view. In general, interventions based on mHealth can improve the breastfeeding status.

## Appendix

Multimedia Appendix 1 Search strategy of the databases.

Multimedia Appendix 2 Forest plots of subgroup analysis.

## Abbreviations

OR: odds ratio
MD: mean difference
mHealth: mobile health
RCT: randomized controlled trial
WHO: World Health Organization
