# Supplementary material for: The Value of Mobile Health in Improving Breastfeeding Outcomes Among Perinatal or Postpartum Women: Systematic Review and Meta-analysis of Randomized Controlled Trials
Source: JMIR Mhealth Uhealth. 2021 Jul 16;9(7):e26098. doi: 10.2196/26098 (PMC8325083; doi:10.2196/26098)
Supplement: Multimedia Appendix 1 [file mhealth_v9i7e26098_app1.docx]

**1. PubMed**

#1“exclusive breastfeeding”[title/abstract]

#2“breastfeeding”[title/abstract]

#3 “Mobile Applications”[MESH]

#4 “Telemedicine”[MESH]

#5 “Text Messaging”[MESH]

#6 “Cell Phone”[MESH]

#7 “Smartphone”[MESH]

#8“mobile” OR “Portable Software Application” OR “Tele*” OR “mHealth” OR “eHealth” OR “e-health” OR “m-Health” OR “?phone*” OR “Text*” OR “Short Message” OR “SMS” OR “app” OR “apps” OR “app-based” OR “electronic” OR “Message*” OR “web” OR “web-based” OR “Internet*” OR “digital*”

#9(randomized controlled trial[pt] OR controlled clinical trial[pt] OR randomized[tiab] OR placebo[tiab] OR clinical trials as topic[mesh:noexp] OR randomly[tiab] OR trial[ti]) NOT (animals [mh] NOT (humans [mh] AND animals[mh]))

#10 #1 OR #2

#11 #3 OR #4 OR #5 OR #6 OR #7 OR #8

#12 #9 AND #10 AND #11

**2. Web of Science**

#1“breastfeeding” OR “exclusive breastfeeding”

#2“Mobile Applications” OR “Telemedicine” OR “Text Messaging” OR “Cell Phone” OR “Smartphone” OR “mobile” OR “Portable Software Application” OR “Tele*” OR “mHealth” OR “eHealth” OR “e-health” OR “m-Health” OR “?phone*” OR “Text*” OR “Short Message” OR “SMS” OR “app” OR “apps” OR “app-based” OR “electronic” OR “Message*” OR “web” OR “web-based” OR “Internet*” OR “digital*”

#3 TS= clinical trial* OR TS=research design OR TS=comparative stud* OR TS=evaluation stud* OR TS=controlled trial* OR TS=follow-up stud* OR TS=prospective stud* OR TS=random* OR TS=placebo* OR TS=(single blind*) OR TS=(double blind*)

**3. Cochrane Library**

#1“exclusive breastfeeding”

#2breastfeeding[MESH descriptor]

#3 #1 OR #2

#4 “Mobile Applications”[MESH descriptor]

#5 Telemedicine[MESH descriptor]

#6 “Text Messaging”[MESH descriptor]

#7 “Cell Phone”[MESH descriptor]

#8 Smartphone[MESH descriptor]

#9 mobile OR “Portable Software Application” OR Tele* OR mHealth OR eHealth OR e-health OR m-Health OR ?phone* OR Text* OR “Short Message” OR SMS OR app OR apps OR “app-based” OR electronic OR Message* OR web OR “web-based” OR Internet* OR digital*

#10 #4 OR #5 OR #6 OR #7 OR #8 OR #9

#11 #3 AND #10

**4. Embase**

#1'breastfeeding'/exp OR 'exclusive breastfeeding'/exp

#2'mobile application'/exp OR 'telemedicine'/exp OR 'text messaging'/exp OR 'mobile phone'/exp OR 'smartphone'/exp

#3'mobile':ti,ab,kw OR 'portable software application':ti,ab,kw OR 'tele*':ti,ab,kw OR 'mhealth':ti,ab,kw OR 'ehealth':ti,ab,kw OR 'e-health':ti,ab,kw OR 'm-health':ti,ab,kw OR '?phone*':ti,ab,kw OR 'text*':ti,ab,kw OR 'short message':ti,ab,kw OR 'sms':ti,ab,kw OR 'app':ti,ab,kw OR 'apps':ti,ab,kw OR 'app-based':ti,ab,kw OR 'electronic':ti,ab,kw OR 'message*':ti,ab,kw OR 'web':ti,ab,kw OR 'web-based':ti,ab,kw OR 'internet*':ti,ab,kw OR 'digital*':ti,ab,kw

#4 #2 OR #3

#5 #1 AND #4

#6 ('crossover procedure':de OR 'double-blind procedure':de OR 'randomized controlled trial':de) AND or  AND 'single-blind procedure':de OR (random*:de,ab,ti AND or :de,ab,ti AND factorial*:de,ab,ti) OR crossover*:de,ab,ti OR ((cross NEXT/1 over*):de,ab,ti) OR placebo*:de,ab,ti OR ((doubl* NEAR/1 blind*):de,ab,ti) OR ((singl* NEAR/1 blind*):de,ab,ti) OR assign*:de,ab,ti OR allocat*:de,ab,ti OR volunteer*:de,ab,ti

#7 #5 AND #6

1. **CNKI**

(主题=母乳喂养) OR (关键词=纯母乳喂养) AND (主题=移动医疗) OR (关键词=互联网) OR (关键词=短信) OR (关键词=电话) OR (关键词=社交软件) AND (关键词=随机对照试验）

1. **Wan Fang**

 主题:("母乳喂养") or 主题:("纯母乳喂养") and 主题:(移动医疗) or 主题:(互联网) or 主题:(电话、短信、社交软件) and 主题:(随机对照实验)

1. **Vip**

关键词=母乳喂养 OR 关键词=纯母乳喂养 AND 关键词=移动医疗 OR 关键词=互联网 AND 关键词=随机对照试验
