# Supplementary material for: The Value of Mobile Health in Improving Breastfeeding Outcomes Among Perinatal or Postpartum Women: Systematic Review and Meta-analysis of Randomized Controlled Trials
Source: JMIR Mhealth Uhealth. 2021 Jul 16;9(7):e26098. doi: 10.2196/26098 (PMC8325083; doi:10.2196/26098)
Supplement: Multimedia Appendix 2 [file mhealth_v9i7e26098_app2.docx]

**The Forest Plot of subgroup Analysis**


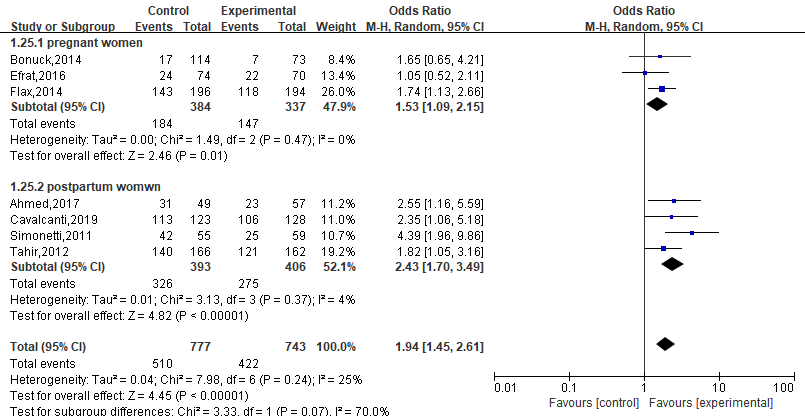


Figure S1. Subgroup Analysis of the Rate of Exclusive Breastfeeding in the First Months after Delivery(participant’s type)


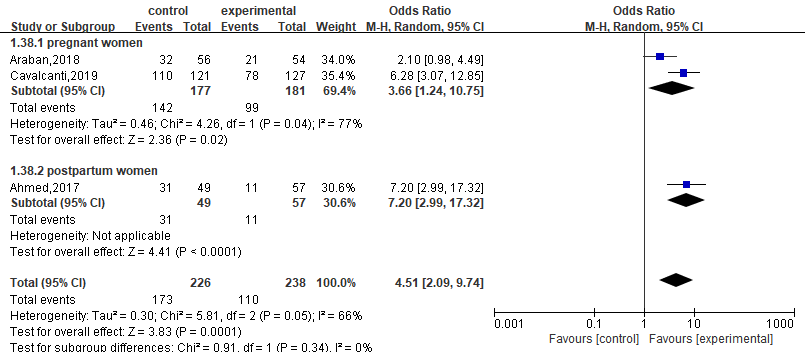


Figure S2. Subgroup Analysis of the Rate of Exclusive Breastfeeding in the Two Months after Delivery(participant’s type)


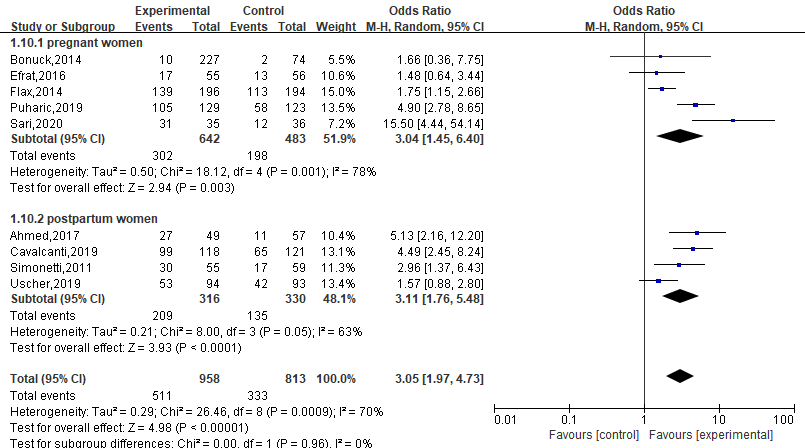


Figure S3. Subgroup Analysis of the Rate of Exclusive Breastfeeding in the Three Months after Delivery (participant’s type)


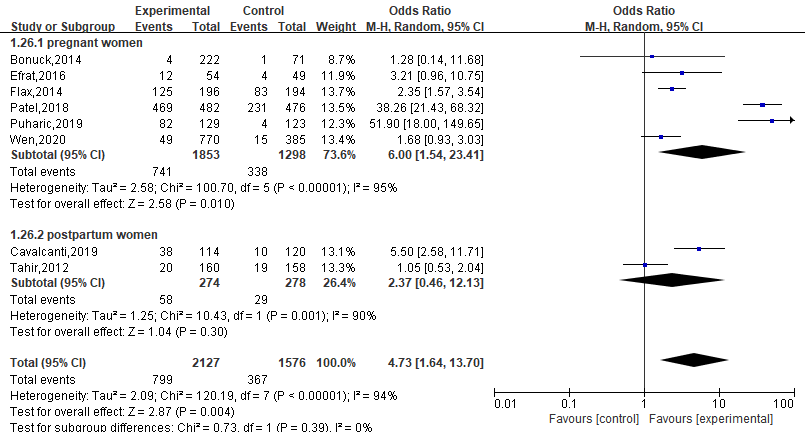


Figure S4. Subgroup Analysis of the Rate of Exclusive Breastfeeding in the Six Months after Delivery(participant’s type)


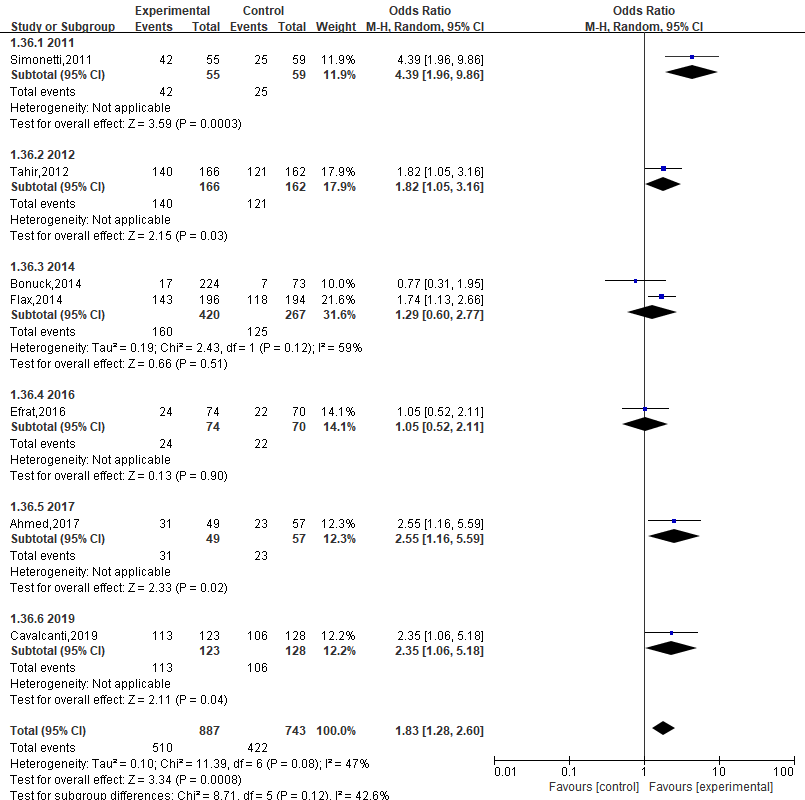


Figure S5. Subgroup Analysis of the Rate of Exclusive Breastfeeding in the First Months after Delivery (publication time)


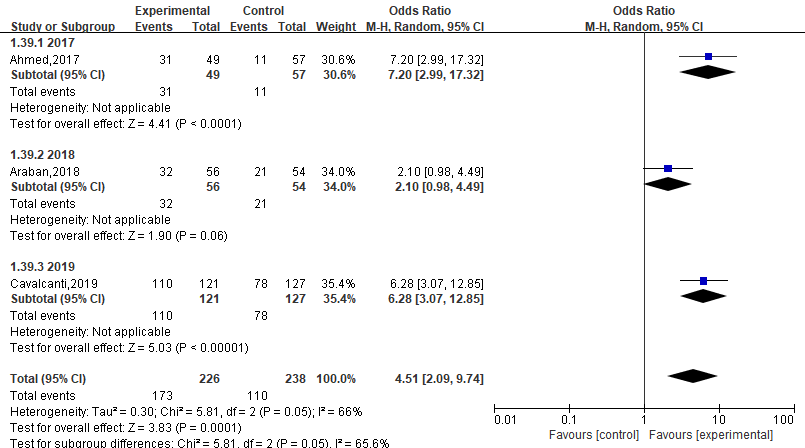


Figure S6. Subgroup Analysis of the Rate of Exclusive Breastfeeding in the Two Months after Delivery(publication time)


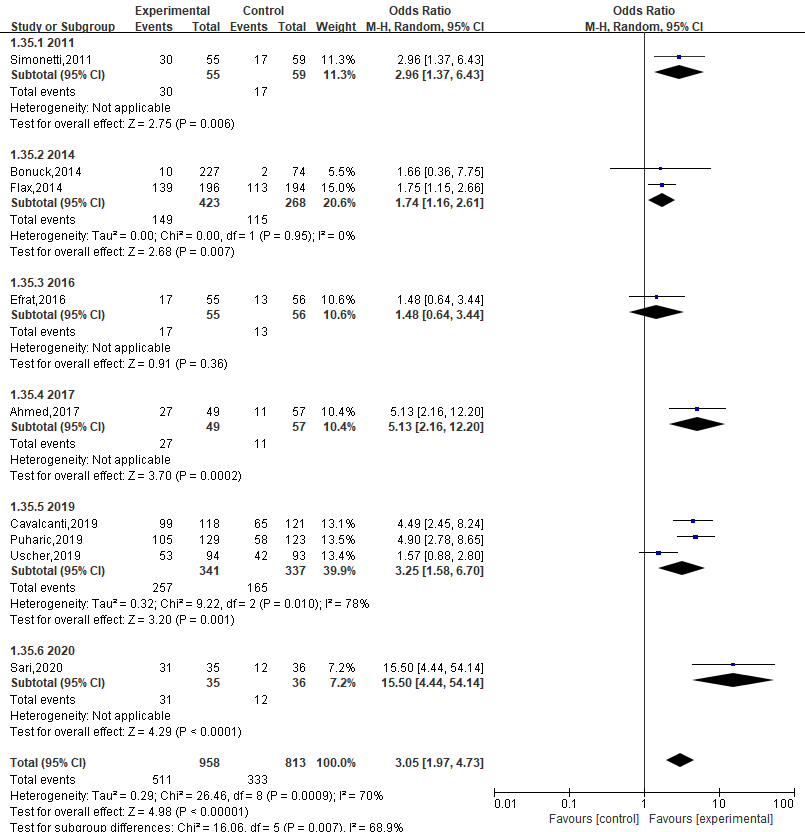


Figure S7. Subgroup Analysis of the Rate of Exclusive Breastfeeding in the Three Months after Delivery (publication time)


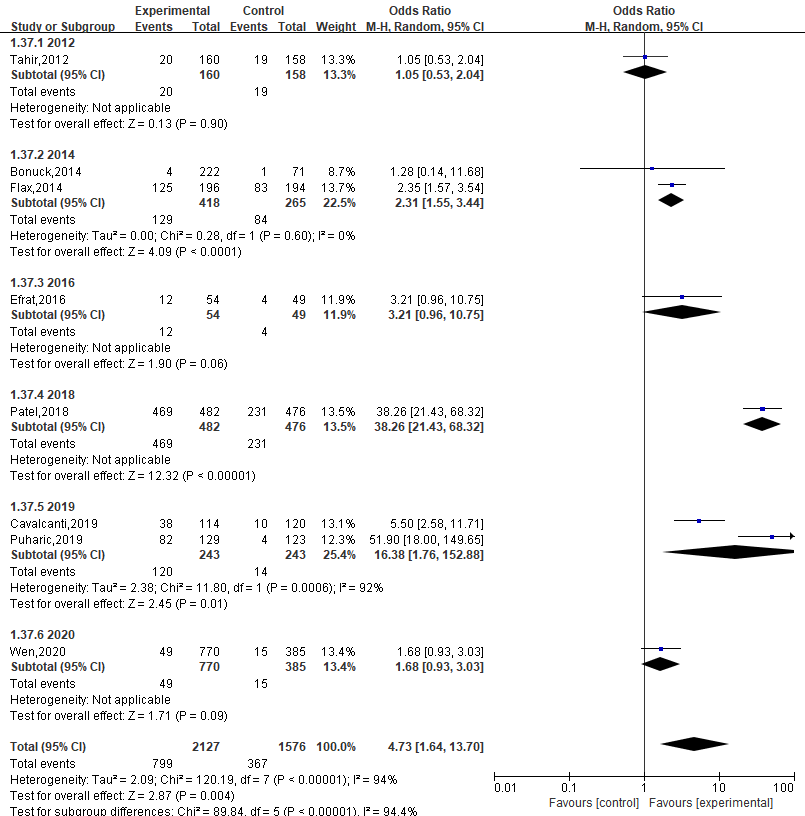


Figure S8. Subgroup Analysis of the Rate of Exclusive Breastfeeding in the Six Months after Delivery(participant’s type)
